# Job satisfaction trends during nurses' early career

**DOI:** 10.1186/1472-6955-7-7

**Published:** 2008-06-05

**Authors:** Trevor Murrells, Sarah Robinson, Peter Griffiths

**Affiliations:** 1 National Nursing Research Unit, King's College London, Florence Nightingale School of Nursing and Midwifery, 57 Waterloo Road, London SE1 8WA, UK

## Abstract

**Background:**

Job satisfaction is an important component of nurses' lives that can impact on patient safety, productivity and performance, quality of care, retention and turnover, commitment to the organisation and the profession. Little is known about job satisfaction in early career and how it varies for different groups of nurses. This paper investigates how the components of job satisfaction vary during early career in newly qualified UK nurses.

**Methods:**

Nurses were sampled using a combined census and multi-stage approach (n = 3962). Data were collected by questionnaire at 6 months, 18 months and 3 years after qualification between 1998 and 2001. Scores were calculated for seven job satisfaction components and a single item that measured satisfaction with pay. Scores were compared longitudinally and between nursing speciality (general, children's, mental health) using a mixed model approach.

**Results:**

No single pattern across time emerged. Trends varied by branch and job satisfaction component. Rank order of job satisfaction components, from high to low scores, was very similar for adult and child branch nurses and different for mental health. Nurses were least satisfied with pay and most satisfied with relationships at 6 and 18 months and with resources (adult and child) and relationships (mental health) at 3 years. Trends were typically upwards for adult branch nurses, varied for children's nurses and downwards for mental health nurses.

**Conclusion:**

The impact of time on job satisfaction in early career is highly dependent on specialism. Different contexts, settings and organisational settings lead to varying experiences. Future research should focus on understanding the relationships between job characteristics and the components of job satisfaction rather than job satisfaction as a unitary construct. Research that further investigates the benefits of a formal one year preceptorship or probationary period is needed.

## Background

Job satisfaction is an important component of nurses' lives that can impact on patient safety, staff morale, productivity and performance, quality of care, retention and turnover, commitment to the organisation and the profession with additional replacement costs (e.g. agency staff) and further attempts to hire and orientate new staff [1]. Turnover rates of 35% to 55% in first year of employment have been reported in the US [2]. In the UK nursing employment fell to 82% 3 years after qualification in a longitudinal study of early career nurses [3]. The cost of a US graduate nurse who leave nursing within 1 year of qualification has been estimated at $40 K [4]. It has been shown that when job satisfaction increases turnover decreases (e.g. [5]). Job satisfaction is dynamic and can vary according to individual characteristics, expectations, style of management, changes to policy and individual lifestyle choices [6-8]. Ensuring that needs of nurses are met is particularly important during early career since what is laid down here could impact dramatically on nurses' contribution in the longer-term.

This paper reports on research that investigated how the components of job satisfaction vary during early career in newly qualified UK nurses using an instrument recently developed for nurses in early career that was valid and reliable across specialty and time.

Interpretation of findings draws on previous research to explain why particular trends may have emerged. This research provided a longitudinal perspective to the understanding of nurses' job satisfaction. Past research has often been cross-sectional, focusing on all types of nurses at all career stages rather than on nurses specifically in early career across different specialties. The few studies on early career have all have focused on the first year or eighteen months [2,4,9]. This study therefore makes a distinctive contribution to the study of nurses' job satisfaction over a longer period of time than previously studied.

### Theories of job satisfaction

Understanding what motivates workers and how this impacts on performance has always interested organisations and managers and different theories have sought to answer this question. Fung-Kam [10] identified four general theories: need/value fulfilment theory; person-environment (P-E) fit theory, the theory of career and the theory of work adjustment. Similarly Adams and Bond [11] classified job satisfaction theories into three groups: discrepancy theories, which examine the extent to which employees needs are satisfied in the work place; equity theories, which highlight social comparisons in the evaluation of job rewards; and expectancy theories which focus on employee motivations. The theory of need/value fulfilment proposes that the discrepancy between individual needs and the extent to which the job meets these needs is negatively related to job satisfaction and expectation gaps [12] have been linked to the violation of the psychological contract between employer and employee [13]. Theory of P-E fit suggests there are person characteristics that suit working environments better than others and working environment characteristics that suit certain individuals better. Drawing on both theories failure to meet expectation has been shown to be related to lower work commitment of graduates and the extent they 'fitted' in was a central motivation to remain with an employer [14]. Connected to P-E fit is the theory of work adjustment. This theory is concerned with the degree of correspondence between individuals and their work environments. Hackman and Lawler [15] believed that the employees' perception of their job rather than the jobs objective characteristics was a more important determinant of job satisfaction. Holland's theory of career [16,17] suggests that personality and type of work are congruent so similar types of individuals converge on the same occupations.

The two theories that have been important in the development of an understanding of job satisfaction in nursing are Maslow's human needs theory [18] and Herzberg and Mausner's motivation-hygiene theory [19]. Maslow identified two types of needs; deficiency needs (physical, safety and belonging) and growth needs (self-actualization and self esteem) [20]. Herzberg and Mausners' theory consists of intrinsic factors or 'motivators' that promote job satisfaction and extrinsic factors or 'hygiene factors' that cause dissatisfaction [8]. Kramer's reality shock theory [21] is based on the reaction new nurses feel once they enter a work situation that they are unprepared for and has also been used to understand job satisfaction in nurses in early career.

### Determinants of satisfaction

Blegen [6] synthesised findings from 48 studies and identified thirteen variables that were most strongly associated with job satisfaction. These included stress, commitment, communication (with supervisor and peers), autonomy (and locus of control), recognition, routinization, and fairness. A secondary analysis of data [22] from the 1977 Quality of Employment Survey [23] found that task variety, relations with co-workers, financial rewards and age were all positively associated with job satisfaction. Conversely role conflict and tenure had negative effects although the latter finding was not consistent with other literature [22]. Work attitudes (supervisor support, work-group cohesion, variety of work, autonomy, organizational constraint, promotional opportunities, work and family conflict, and distributive justice) were also important in explaining the job satisfaction of registered nurses in the United States [24].

Different managerial styles and practices at the organisational unit level (e.g. ward) can have a direct bearing on nurse satisfaction. The work of Adams and Bond [11] highlighted the importance of interpersonal relationships with nurses and other medical staff, workload and ward cohesiveness. A number of studies [25-28] have shown a positive association between autonomy and levels of job satisfaction and which has been confirmed amongst nurses [6,10,29-31].

The effect of educational level on job satisfaction has been conflicting. Some studies have found a positive association with job satisfaction [32,33] and others a negative association [3,6,34]. Lower level qualifications impacted positively on job satisfaction based on findings from a survey of NHS nurses [35]. The same study found training had a positive impact on job satisfaction but diminished with the number of training spells. The inverse relationship supports the argument that education raises expectations that subsequently are not met [14] whereas a positive correlation suggests that the greater extrinsic rewards that come with education raises satisfaction. Blegen [6] found that job satisfaction correlated less strongly with age or years of experience, while Shields and Ward [35] found that increasing age, marriage, and children impacted positively on nurses' satisfaction.

In a study of nurses working in the NHS [35], individuals who stressed non-pecuniary reasons (e.g. flexibility of hours, helping others) had significantly higher job satisfaction than those who did not (e.g. attracted by job security, promotion prospects, pay). Not being graded fairly was the largest negative determinant of overall job satisfaction and not having the hours to suit an individual's preference had a negative impact. Absolute and relative levels of pay (compared with other occupational groups) are also important [36].

### Job satisfaction in early career

There have been a number of US studies recently which have researched job experience and satisfaction during the early years after qualification [1,2,4,5,9]. These have covered the period up to 18 months after qualification and some are longitudinal [2,4,9]. Graduate nurses were found to lack confidence in skill performance and had concerns with peer and preceptor relationships, dependence (on others) and becoming an independent practitioner, the work environment, organizational and priority setting and communications with physicians [9]. Low-points in satisfaction and confidence between 6 and 12 months have been reported in the US [9]. Graduates participating in residency programs were particularly vulnerable between entry and 6 months [2]. Dissatisfaction with patient care, scheduling (work-life balance) and pay may precipitate job exit [4]. Low pay satisfaction has been reported in the US [9].

Before a nurse can practice in the UK they must hold a bachelors degree or diploma in nursing. These educational programmes consist of theory and practice (in community and hospital settings) in roughly equal proportions beginning with a common foundation programme (CFP) lasting 12 months followed by about two years in one of the four branches of nursing: adult(general), mental health, learning disabilities or children's nursing. Either qualification allows a nurse to register with the Nursing and Midwifery Council (formally the United Kingdom Central Council (UKCC)). The diploma qualification is similar in educational level to a US associate degree but training extends over three years and there is a single level of professional registration (equivalent to a US Registered Nurse) irrespective of the training programme. Adult nurses work with adults of all ages and children's nurses with newborns to adolescent (0–16 years). Both provide care to patients with chronic and acute health conditions. Learning disability nurses help, care and develop the skills of people with disabilities in family, community and residential settings, and adult and young peoples' education. Mental health nurses provide care for mentally ill people and their families primarily in the community and less often in a hospital setting. Nurses can specialise further after gaining experience in more general settings post-registration. At the time of the study the pre-registration degree and diploma were the only points of entry. The majority (over 80%) of individuals opted for the diploma programme.

Newly qualified nurses in the UK typically used to start as D Grade nurses and after a minimum of 6 months post-registration experience could become E grade nurses. Nurses at this grade were often encouraged to gain valuable management experience and/or receive further training in a specialty (e.g. accident and emergency). F grade nurses had more of a managerial role and were sometimes left in charge of a ward or other setting. In December 2004 these grades were superseded in the UK by Agenda for Change pay bands [37].

Our research examines how job satisfaction varies amongst newly qualified nurses in the England over time in early career (6 months, 18 months, 3 years) and to what degree trends in factors arising from a factor analysis of a multi-item job satisfaction question [38] vary between specialisms.

## Methods

### Design

The design was longitudinal and correlational with data collected at four time-points (qualification, 6 months, 18 months, 3 years). The 6 month time-point was selected to reflect early experiences, 18 months to reflect established early experiences and first promotion, and three years to reflect consolidation. More frequent follow-up was considered but deemed undesirable due to the potential burden this would place on respondents who were asked to complete large questionnaires at each survey sweep.

### Sample

The study population was formed from all nurses qualifying in 1997/98 from the diploma programme in England. A mixed approach to sampling was undertaken determined by the population size of each branch. Initial estimates of the children's and learning disability branch nurse populations were small (574 and 246 respectively). Estimates of the adult and mental health branches were much larger (4850 and 1142 respectively). A decision was taken therefore to sample all children's and learning disability nurses qualifying in England. The larger adult and mental health branch nurse populations were sampled using a multistage approach based on the eight regional health authorities (that existed at the time), colleges and intakes. Between three and eight college of nursing (depending on branch) were located in each region. Approximately a half and two-thirds of colleges were sampled from each region for the adult and mental health branches respectively. These sampling fractions were based on information previously collected from the English National Board and our own enquiries to Colleges of Nursing. There was further sub-sampling at the college level of the adult branch when intakes were large. The number of college intakes(classes) for the adult, children's nurses, mental health and learning disability branches was 46, 49, 56 and 34, the total number eligible to be recruited was 2109, 758, 293 and 802 respectively (total 3962) and recruitment rates were 87%, 93%, 90% and 85% respectively (88% overall). A comprehensive report of the sampling strategy is found in Marsland and Murrells [39].

The number and percentage responding at each sweep was as follows: qualification (3009, 80%), 6 months (2524, 64%), 18 months (2118, 53%), three years (1785, 45%). Non-response is common to most longitudinal postal surveys particularly when the point of contact is home address. We asked nurses to inform us of address changes. Questionnaires were always sent to last known address and if no response to second address (typically parents) and finally to the UKCC registered address (written on the envelope at the UKCC premises). Higher non-response rates were noted in parts of the country where nurses were likely to change address more often (e.g. London) and therefore contact was more easily lost. Region of workplace was therefore included in the statistical model to compensate for regional heterogeneity.

### Job satisfaction measure

We wanted to measure job satisfaction specifically for nurses in early career. Existing nursing scales suffer from a number of limitations. Some have not been adequately tested for reliability and validity, they are often very long or very short, not contemporary, developed from theory without contextual representation and developed on nurses from different healthcare systems [40]. For this reason we chose to develop a new instrument [40] using the five-step method recommended by Spector [41]. Following in-depth interviews of 30 diploma-qualified nurses a pool of items was generated and the number of interviewees who regarded each item as important was noted. A total of 34 items were identified. A small number of items were added as the study progressed to reflect aspects pertinent to career after qualification. Each item was measured on a five point scale from *very satisfied *to *very dissatisfied*. Some items had a 'not applicable' or similar response option. The set of items available for psychometric analysis was reduced because either the item did not apply (e.g. many respondents did not have a family or partner) or the item did not apply across all time points (e.g. content of appraisals). Further analysis was confined to the twenty items applicable to over 90% of respondents. Psychometric analysis was initially confined to the adult branch and included tests of temporal stability across time. Further validation across specialties and over time has now taken place [38]. Factor analysis (not reported here) of twenty selected items that were asked at all three time-points and were applicable to at least 90% of respondents identified two potential seven factor measurement models (Client Care, Staffing, Development, Relationships, Education, Work-Life Interface, Resources) for nurses' job satisfaction in early career that differed on the loading of one item, *emotional support from immediate line-manager *either on the *Relationship*(Model I) or the *Development *(Model II) factor. There was little difference in the fit of the two models. Model I was a better fit for the adult branch and model II for the child and mental health branches. The difference in overall fit however was small (Root Mean Square Error Approximation 0.021 and 0.022 respectively). We did not want to further burden respondents who were asked to complete a large questionnaire (sometimes running to over 60 questions) on four occasions over a three year period) with an additional questionnaire to test inter-rater reliability however internal consistency was good with Cronbach's alphas all above 0.7 except for *Work-Life Interface *and correlations between factor scores recorded at 6 and 18 months ranged from 0.27 to 0.42. The instrument did not provide a good fit for the learning disability nurses and for this reason we have excluded this branch from the data analysis. Findings were similar for both models and for this reason (and for brevity) we report on the second measurement model (Table 1) which had a better fit for two of the three remaining branches.

**Table 1:** Measurement Model

**Factor**	**Item**
**Client Care**	Proportion of time I spend/spent providing direct client care ('hands on' care)
	Opportunities to provide good quality care
	Proportion of time I spend/spent on paperwork
**Staffing**	Ratio of qualified to unqualified staff on days
	Number of staff usually on days
**Development**	Opportunity to reflect on my practice with someone of a higher grade/position
	Opportunity to reflect on practice with a group of colleagues
	Opportunity to reflect on my own practice on my own while at work
	Frequency of discussions about developing my career
	Constructive feedback on my work from staff of a higher grade/position
	Emotional support from my immediate line manager
**Relationships**	Quality of working relationships with colleagues
	Emotional support from nurses of the same grade/position
**Education**	Opportunitiy to go on courses other than study days/workshops
	Opportunity to go on study days/workshops
**Work-Life Interface**	Notice of off duty
	Combining work hours with social life
	Frequency with which I leave work on time
**Resources**	
*Adult and Child*	Availability of equipment(e.g. hoists)
	Availability of supplies (e.g. dressings)
*Mental Health*	Availability of equipment (e.g. audiovisual, art materials, books)
	Availability of facilities (e.g. day room, quiet room, interview room)
**Pay**	Pay in relation to level of responsibility

Pay which was represented by a single question item was included as a notional eighth factor. The job satisfaction question included two items on pay and grade which were *Pay in relation to responsibility *and *Grade/position in relation to level of responsibility*. The second item was asked from 18 months onwards and was therefore excluded from the psychometric analysis on the grounds that it had not been asked across all three time-points.

### Access, participation and data collection

All participants gave informed consent. An initial request to access students was made by letter to the 'head' of each college [42]. The letter described the project and asked if research team members could meet with students and invite them to participate. The letter emphasised that participation was voluntary. The recruitment meeting consisted of a short presentation of 10 minutes followed by a discussion of up to 30 minutes. During the meeting forms were circulated and completed by those willing to participate. Information requested included current address, a second, more permanent, address (e.g. parents). The form was accompanied by an information sheet. Various strategies were used to recruit non-attendees such as asking group members and course leaders to pass on recruitment packs, writing to non-attendees via the course leader and repeating the visit when there were a large number of non-attendees.

Agreement to participate was high for those who attended these meetings (over 80%) but on the few occasions when face-to-face recruitment was not possible this fell to below 50%. Participants were supplied with change of address slips (and freepost envelopes) so if they moved we were kept informed. Change of address slips were also supplied with each survey questionnaire. Between 18 months and 3 years after qualification lost participants were traced via the UKCC.

Job satisfaction information was collected as part of a much larger postal questionnaire sent at 6 months, 18 months and 3 years after qualification. Baseline demographics and other profile information were collected at qualification. A question was designed specifically to collect career history information and some of this information (e.g. number of previous nursing posts) was used in the modelling.

### Ethical Considerations

Although the longitudinal study of nurses qualifying from the pre-registration diploma course predated the requirement of Multi-Centre Research Ethics Committee approval, guidance was followed from staff of the university from which students were recruited as to the internal procedures required for ethical approval.

### Data Analysis

Factor scores for each nurse were produced by taking the mean of all the non-missing item scores. We applied the more stringent condition that at least half of each factor's items had to be answered otherwise the factor score was set to missing. There is not set guideline on this. Bryman and Cramer [43] used 50% or more as their exclusion criteria in the example they presented. We could have considered multiple imputation [44] but the success of this method depends very much on the correction specification of the non-response model and so this option was not pursued. The average percentage of respondents answering all items of a factor was 94% therefore any resulting biases should be small.

A comparison of previous satisfaction levels of responders and those who did not respond at subsequent time points was conducted in order to determine if non-response was itself related to satisfaction. Response group means were compared statistically using ANOVA. This analysis did not reveal any major differences in job satisfaction scores between response groups nor did an analysis confined to those nurses with complete data across all three time-points differ from nurses who provided data at one or more time-points. On the few occasions where differences did emerge these have been identified in the tables showing factor means.

Summary statistics (means, standard errors) were produced, by branch, across the three time-points. A strategy for fitting repeated measures models, similar to the one proposed by Wolfinger and Chang [45] was followed. Three possible covariance structures (compound symmetry, Huynh-Feldt and unstructured) were tested statistically and the best fitting covariance structure was selected. Differences between branches at each time-point were tested statistically using ANOVA. The F-test for the time effect within branch was obtained from a mixed model incorporating the selected covariance structure. In the tables showing factor means we test the equality of branch means at each time-point and show the result in the far right hand column. At the foot of each factor sub-column we test the equality of means across the three time-points within branch. At the end of the same row is a test of equality of means across branch and time as indicated by the four degrees of freedom in the numerator of the F-test. All these tests were computed using SAS Version 8.

The analysis across time within branch was then repeated having accounted for variation, attributable to ten moderating variables. Some were time varying (children, spouse or partner, job grade, region, age, number of previous nursing posts, time in current job) whilst others did not change across time (sex, ethnicity, highest education qualification) (Table 2). All these variables were entered into the model along with a factor representing the three time-points.

**Table 2:** Sample profile

	**Adult**			**Child**			**Mental Health**		
**Profile variable**	**6 months **(1338)	**18 months **(1117)	**3 years **(901)	**6 months **(558)	**18 months **(477)	**3 years **(373)	**6 months **(442)	**18 months **(365)	**3 years **(300)
	Mean	Mean	Mean	Mean	Mean	Mean	Mean	Mean	Mean
**Age**^†^	27.4	28.5	29.8	24.8	25.8	27.3	30.1	31.1	32.8
	%	%	%	%	%	%	%	%	%
**Female**	93.4	94.1	94.4	95.2	85.2	94.9	70.0	72.7	75.0
**Ethnic Group**									
White British	87.1	88.8	90.0	91.6	93.1	92.8	80.6	83.4	86.4
White Irish	5.4	4.2	3.3	2.3	1.7	1.6	5.7	4.8	3.7
Other White	2.9	2.9	2.2	2.5	2.3	2.4	3.7	3.9	3.4
Asian, Black, Chinese	4.1	3.4	3.6	2.9	2.1	2.1	9.6	7.4	6.2
**Highest Educational Qualification**									
Degree	4.5	4.6	4.8	3.1	2.5	2.1	15.9	14.9	16.0
Sufficient for degree entry	24.9	26.1	27.4	32.6	32.1	34.9	22.8	23.3	23.3
Not sufficient for degree entry	41.3	41.6	42.8	44.3	44.4	41.6	34.4	37.0	37.0
Access course/DC test	18.6	17.5	15.6	8.2	8.6	9.9	18.7	18.3	17.4
Other	10.7	10.2	9.5	11.8	12.4	11.5	8.3	6.6	6.3
**Children living with respondent**^†^	22.4	28.0	33.2	10.6	13.6	18.5	28.5	32.7	37.5
**Spouse or Partner**^†^	68.7	72.8	77.6	59.8	68.1	71.1	65.6	74.0	76.1
**Region**^†^									
London	12.1	9.5	8.8	26.0	25.0	24.9	15.3	14.5	12.1
South East	18.4	17.5	14.7	17.1	14.3	12.6	21.0	18.6	18.5
South West	9.5	9.1	9.8	5.7	6.5	7.2	9.0	10.4	9.6
West Midlands	8.8	8.8	8.3	12.2	12.2	12.1	8.4	10.3	10.2
Eastern	10.2	10.6	10.4	5.3	6.5	7.5	9.8	9.1	10.6
Trent	9.5	9.4	9.5	6.3	7.6	8.0	8.8	8.0	7.3
North West	14.9	13.3	13.8	15.5	13.4	11.5	10.3	9.5	9.1
Northern & Yorkshire	15.4	14.7	13.6	10.9	10.5	8.9	15.7	15.9	15.0
Other/Not Nursing	1.3	7.3	11.2	1.1	4.2	7.2	1.7	3.8	7.6
**Nursing grade**^†^									
D or Lower	99.6	73.2	38.0	99.5	59.5	20.4	93.7	23.8	8.6
E	0.1	11.0	40.0	0.2	31.9	57.4	3.7	63.5	50.0
F or higher	0.0	0.7	3.3	0.0	0.4	5.1	0.0	3.6	24.5
Other/Not Nursing	0.3	15.2	18.6	0.4	8.2	17.2	2.6	9.0	16.9
	Mean	Mean	Mean	Mean	Mean	Mean	Mean	Mean	Mean
**Number of nursing posts**^†^	n/a	1.6	2.4	n/a	1.6	2.4	n/a	2.1	2.9
**Time in current nursing job**^†^	5.5	10.2	11.2	5.6	10.3	10.3	5.3	8.7	12.2


† time varying variable

## Results

### Sample Profile

The sample profile is shown in Table 2.

Mental health nurses were older at qualification than adult branch nurses by about two and a half years who were themselves older than child branch nurses also by two and a half years. Over 90% of adult and child branches were female compared with about 70% of mental health nurses. Mental health nurses were more likely to belong to an ethnic minority than the other two branches and already have a degree level qualification. Grade progression occurred more rapidly for mental health nurses whereas progression was slowest for adult branch nurses. By 3 years most nurses were onto their second or third post. Nurses tended to stay in post for similar amounts of time and by three years had been in their current post for almost a year.

### Job satisfaction trends, six months to three years post-qualification

For *Client Care *(Table 3), scores were highest for the children's nurses and lowest for mental health nurses. Scores changed little across time in both cases. The time profile was different for the adult branch with a similar score to the mental health branch (3.18 vs. 3.13) at 6 months rising to 3.44 at 3 years. Children's nurses were on the whole happier with *Staffing *(Table 3) than the other two branches. Satisfaction with *Staffing *dipped at 18 months for children's nurses and mental health nurses whereas a linear upward trend was observed for adult branch nurses. For these nurses a low score (3.12) was obtained at 6 months and by 3 years adult branch nurses were more satisfied with *Staffing *than mental health nurses.

**Table 3:** Factor means for Client Care, Staffing, Development and Relationships by branch and time

		**Adult**			**Child**			**Mental Health**			Branch comparison (F [df_n_, df_d_] p)
Factor	Time-point	No.	Mean	SE	No.	Mean	SE	No.	Mean	SE	
**Client Care**	6 months	1255	3.18	0.03	533	3.54	0.04	427	3.13	0.05	(31.75 [2,2325] < .001)
	18 months	942	3.37	0.03	446	3.51	0.04	336	3.14	0.05	(16.27 [2,2325] < .001)
	3 years	755	3.44	0.04	326	3.53	0.05	255	3.03	0.06	(24.34 [2,2325] < .001)
	(F [df_n_, df_d_] p)	(23.26 [2,1322] < .001)			(0.33 [2,558] .72)			(1.75 [2,568] .17)^1^			(8.55 [4,2325] < .001)
**Staffing**	6 months	1241	3.12	0.03	529	3.55	0.04	414	3.41	0.05	(32.17 [2,2301] < .001)
	18 months	909	3.27	0.04	435	3.39	0.05	305	3.24	0.06	(2.60 [2,2301] .075)
	3 years	711	3.41	0.04	307	3.51	0.05	213	3.32	0.07	(2.26 [2,2301] .10)
	(F [df_n_, df_d_] p)	(17.28 [2,1312] < .001)			(3.92 [2,715] .020)^2^			(2.67 [2,491] .070)^1^			(8.15 [4,2301] < .001)
**Development**	6 months	1257	3.05	0.03	534	3.23	0.04	427	3.30	0.04	(16.14 [2,2327] < .001)
	18 months	942	3.18	0.03	446	3.18	0.04	336	3.34	0.05	(4.48 [2,2327] .011)
	3 years	755	3.16	0.03	326	3.20	0.05	255	3.32	0.06	(2.51 [2,2327] .081)
	(F [df_n_, df_d_] p)	(9.45 [2,1323] < .001)			(0.53 [2,559] .59)			(0.26 [2,568] .77)^1^			(2.87 [4,2327] .022)
**Relationships**	6 months	1257	4.19	0.02	535	4.32	0.03	425	4.17	0.04	(6.19 [2,2326] .002)
	18 months	938	4.19	0.03	446	4.32	0.03	335	4.14	0.04	(7.07 [2,2326] < .001)
	3 years	753	4.16	0.03	324	4.25	0.04	254	4.08	0.05	(3.71 [2,2326] .025)
	(F [df_n_, df_d_] p)	(0.45 [2, 1323] .64)			(1.44 [2,559] .24)			(1.20 [2,565] .30)^1^			(0.28 [4,2326] .89)

Unstructured covariance unless indicated by superscript ^1 ^Compound symmetry; ^2^Huynh-Feldt

*Development *scores (Table 3) remained reasonably stable across time for all branches. The adult branch had a significantly lower score at 6 months but had caught up children's nurses by 18 months.

The only factor to produce scores in excess of 4 across all branches and time-points was *Relationships *(Table 3). Trends were stable across time and were significantly higher for children's nurses than the other two branches although in real terms the differences were small.

A common profile for *Education *scores (Table 4) emerged for all branches starting off low at 6 months followed by a sharp increase at 18 months where it remained, apart from a small increase, for children's and mental health nurses whereas the score continued on an upward trajectory for the adult branch. By 3 years the adult branch nurses had almost caught up the mental health nurses.

**Table 4:** Factor means for Education, Work-life interface, Resources and Pay by branch and time

		**Adult**			**Child**			**Mental health**			Branch comparison
Factor	Time-point	No.	Mean	SE	No.	Mean	SE	No.	Mean	SE	(F [df_n_, df_d_] p)
**Education**	6 months	1248	3.06	0.04	531	3.36	0.05	425	3.18	0.06	(10.76 [2,2323] < .001)
	18 months	940	3.40	0.04	445	3.66	0.05	334	3.61	0.06	(9.90 [2,2323] < .001)
	3 years	754	3.59	0.04	326	3.70	0.06	255	3.63	0.07	(1.16 [2,2323] .31)
	(F [df_n_, df_d_] p)	(56.22 [2,1321] < .001)			(16.57 [2,558] < .001)			(19.27 [2,444] < .001)			(1.77 [4,2323] .13)
**Work-Life Interface**	6 months	1257	3.37	0.03	533	3.49	0.04	427	3.69	0.04	(18.82 [2,2325] < .001)
	18 months	939	3.46	0.03	446	3.49	0.04	336	3.63	0.05	(4.88 [2,2325] .008)
	3 years	755	3.63	0.03	326	3.59	0.05	256	3.78	0.05	(4.13 [2,2325] .016)
	(F [df_n_, df_d_] p)	(21.06 [2,1322] < .001)			(1.80 [2,558] .17)			(2.97 [2,569] .052)^1^			(2.47 [4,2325] .043)
**Resources**	6 months	1253	3.52	0.03	532	3.73	0.04	427	3.15	0.05	(36.74 [2,2324] < .001)
	18 months	938	3.69	0.03	445	3.69	0.04	333	3.09	0.06	(48.24 [2,2324] < .001)
	3 years	740	3.78	0.03	320	3.80	0.05	254	3.08	0.07	(60.41 [2,2324] < .001)
	(F [df_n_, df_d_] p)	(24.13 [2,1321] < .001)			(2.16 [2,558] .12)			(0.61 [2,565] .54)^1^			(6.46 [4,2324] < .001)
**Pay**	6 months	1254	2.41	0.04	530	2.88	0.05	427	2.55	0.06	(26.06 [2,2321] < .001)
	18 months	933	2.67	0.04	444	2.81	0.06	330	2.66	0.07	(2.29 [2,2321] .10)
	3 years	751	2.63	0.04	326	2.78	0.07	255	2.65	0.07	(2.13 [2,2321] .12)
	(F [df_n_, df_d_] p)	(20.74 [2,1318] < .001)			(1.02 [2,739] .36)^1^			(1.48 [2,563] .23)^1^			(5.56 [4,2321] < .001)

Unstructured covariance unless indicated by superscript ^1 ^Compound symmetry

Results biased by non-response (attrition): Adult, resources; Mental Health, pay

Satisfaction with *Work-Life Interface *(Table 4) progressed positively, although at different rates across all branches. Mental health nurses had established higher levels early on. Greatest progress was observed for the adult branch nurses who started from a significantly lower base at 6 months and had overtaken children's nurses by 3 years.

Satisfaction with *Resources *(Table 4) remained low throughout for mental health nurses whereas satisfaction improved over time for both adult branch and children's nurses although there was a slight dip at 18 months for children's nurses. The scores for the mental health nurses were comparable to that obtained on *Client Care *and it would not be unreasonable to assume that there was connection between the two.

The lowest scoring factor of all was *Pay *(Table 4). *Pay *satisfaction improved over time for the adult branch and after starting off low (2.44) had almost caught up mental health nurses (2.63 vs. 2.65) by 3 years whereas it fell for children's nurses although this was neither statistically significant nor substantial in real terms.

**Figure 1:**
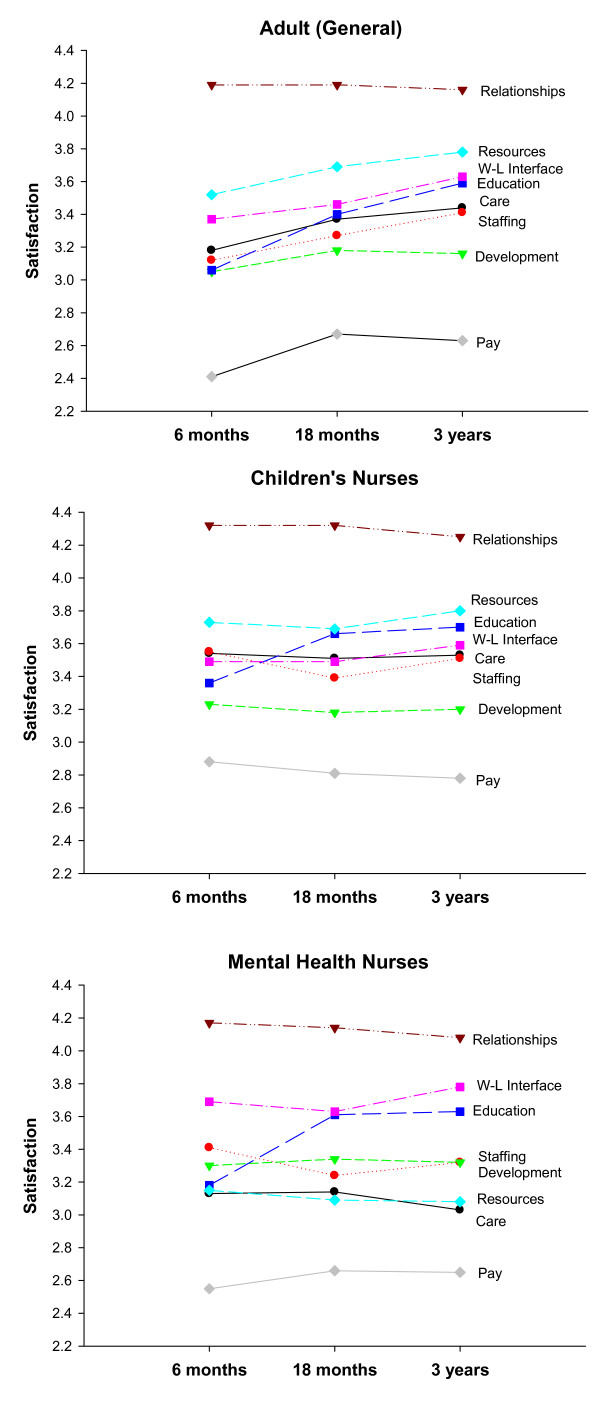
Satisfaction trends.

In summary the strongest trends emerged for the adult branch with increasing job satisfaction from six months onwards for *Client Care*, *Staffing*, *Education*, *Work-Life Interface *and *Resources *(Tables 3 and 4). There were also significant increases between six and eighteen months for *Development *and *Pay*. Fewer significant trends were apparent for the child and mental health branches. Mean profiles over time were found to vary significantly between branches on the *Care*, *Staffing*, *Development*, *Work-Life Interface*, *Resources *and *Pay *factors. All eight factors differed significantly between branches at six months. Six differed significantly at eighteen months and four at three-years. The rank order of job satisfaction components was very similar for adult and child branch nurses (Figure 1).

Means were then adjusted to account for variation attributable to the ten moderating variables. These variables accounted for a small proportion of the variability and findings remained largely unchanged although there was some change in statistical significance (Adult, *Development *and *Resources *no longer significant; Mental health, *Resources *significant downward trend) and more substantively for Children's nurses, *Education *remained significant but the direction of trend changed (6 months: 3.51, 18 months: 3.57, 3 years 3.22)

## Discussion

The confusing picture about the impact of time on satisfaction in the literature is confirmed here. There is no single pattern and the different experiences of the three specialities studied seem to lead to different patterns. Further, the effect of time varies across dimensions of satisfaction. Many of the trends are of little practical significance and some of the differences between branches are small. What does emerge strongly is that recently qualified nurses are not satisfied with their pay (in relation to level of responsibility) which has been reported frequently elsewhere for nurses generally [32,35,36,46-49] while they are highly satisfied with the quality of working relationships and emotional support that they receive from colleagues. This confirms previous research that nurses are satisfied with aspects of support received in their immediate work area but often less satisfied with higher level management and development opportunities [1].

First impressions are positive for the adult branch, where the trends were mostly upwards, mixed for the child branch and a tendency towards the negative for the mental health branch. It would appear that newly qualified adult branch nurses have been able to make the adjustment to work more effectively than the other two branches although their satisfaction levels early on are lower than the other two branches. The decline in satisfaction for mental health nurses suggests that these nurses have perhaps faced the bigger challenge. Satisfaction with client care and resources both start at a low level and remain stubbornly low and it would not be unreasonable to propose that there was casual relationship between the two.

The V-shaped trend reported by William's and colleagues [2] amongst postbaccalaureate nurses undergoing a residency program was found to be consistent with Kramer's theory [21] and a decline in job satisfaction measured using the McCloskey-Mueller Satisfaction Scale was observed between entry and 6 months. Casey and colleagues [9] have suggested that new nurses take at least 12 months to feel comfortable and confident. In this study mean scores were lower at 6 months than either of the two later time-points on all job satisfaction components except *Relationships *for adult branch nurses. Children's nurses had a greater number of lowest scores at 18 months (*Client Care*, *Staffing*, *Development*, *Work-Life Interface*, *Resources*) than at 6 months (*Education*, *Work-Life Interface*) and 3 years (*Relationships, Pay*). Mental health nurses had three lowest scores at 6 months (*Development*, *Education*, *Pay*), one at 18 months (*Staffing*) and four at 3 years (*Client Care*, *Relationships*, *Work-Life Interface*, *Resources*) suggesting that if reality shock is operating its effects happen at different times and probably in different ways depending on the specialism. The adult branch nurses would appear to provide the best fit to the theory. The theory states that satisfaction drops as formal orientation ends and working independently begins [4]. The findings for children's nurses and mental nurses suggest that reality shock may extend beyond the period specified by Kramer [21] and confirmed by Williams [2], or be replaced by another phase which is less about shock and more about realism and coping with additional responsibilities. By 3 years 63% of children's nurses and 75% of mental health nurses were employed as staff or senior nurses and almost 25% of mental health nurses were in senior staff nurse posts. The rapid ascendancy of mental health nurses could be one reason for their lack of upward job satisfaction trends.

The exposure of student nurses today to the nursing environment is different from the past when UK training was hospital based and students were employees of the organisation. Nurses then were perhaps in a better position to adapt, often working in the organisation where they trained and therefore were less likely to suffer from the type of reality shock that newly qualified nurses' encounter today. They may have been better able come to terms with any discrepancies between their own needs and that of the job and organisation prior to qualification allowing for a smoother transition.

There may be a desire for nurses to compare themselves against other graduates and professions. Pay has become a major issue above that of autonomy, flexibility and a supportive organization particularly when there are shortages of nurses, rising levels of acuity and increasing workloads [49]. Satisfaction with *Pay *was low and was the only factor to produce scores consistently below 3. There was some improvement over time for the adult and mental health branch nurses however there was a small non-significant decrease in *Pay *satisfaction for children's nurses. Nurses feel poorly paid compared with other public sector workers [50] but paradoxically the estimated impact of increased wages on nurse retention is potentially small [51]. Children's nurses, because of their young age, may be more prone than older nurses to making comparisons with their peers who on graduating are moving into better paid jobs. The larger the differential between the NHS wage and the outside wage the more likely nurses are to leave [51]. Around 25% of children's nurses work in the London region where higher non-nursing salaried jobs are found. There are constant reminders of city bonuses in the press and media, housing costs are high and it is very difficult for nurses, along with other key workers, to get on the housing ladder. Reasons for poor retention in London include large numbers of young mobile workers, lack of access to affordable child care, high cost of living and heavy workloads [52,53]. Therefore financial considerations will be central to nurses thinking. It has been suggested that more research is required on the effect of new nurses personal and financial stressors [9]. The effect of peer comparison may lessen as the UK higher education sector moves towards even higher levels of participation, more graduates are saddled with debts and there are not the jobs to match the degree qualifications. Grow your own approaches have been suggested as one way of retaining nurses and reflecting the diversity of local populations [53].

In this study pay was measured by only one item. We advise that the excluded item *Grade/position in relation to level of responsibility *be included after a reasonable period of time in work. Other items could be added or alternatively a generic instrument measuring satisfaction with rewards could be used.

Previous research on registered general nurses found that younger nurses were less satisfied with their overall jobs than older nurses [35]. Children's nurses had higher scores on six of the factors suggesting that this specialism may have a counterbalancing effect on age.

The loss of the academic schedule requires a period of adjustment and has described as something akin to a "grieving process" [4]. Satisfaction with *Work-life Interface *is comparatively high for the child and mental health nurses. For adult branch nurses *Work-Life Interface *shows gradual improvement over time from a lower base level. Becoming accustomed to regular shift work and juggling the tensions between work and personal life may have taken longer for these nurses. The management of shifts and schedules during orientation [4] so as not to disaffect newly qualified staff is important. The *Work-Life Interface *factor in this study was limited. It was not possible to include items for combining work hours with life with spouse/partner and responsibilities for children since they did not apply to a sufficient number of nurses. The transitional nature of work and life satisfaction probably requires a more dynamic tool for its measurement than the one used here that goes beyond leaving work on time, notice of duty and social life.

The whole issue of resources, and that includes staffing, will have a direct impact on patient care. It is worth noting for adult branch nurses in this study that when satisfaction with *Resources *and *Staffing *increased so did satisfaction with *Client Care*. Satisfaction with *Staffing *dipped at 18 months for children's nurses however this did not impact on satisfaction with *Client Care *whilst for mental health nurses satisfaction with resources remained consistently low as did satisfaction with *Client Care *and reflects the longstanding perception that mental health services are under resourced in comparison with other services [54,55]. Satisfaction with *Staffing *tracks satisfaction with *Client Care *and supports previous research that has shown that better staffing (e.g. patient-to-nurse ratios) is linked to improved patient outcomes [56,57]. It is well established that high nurse turnover can impact considerably on the well-being of nurses who remain and patient outcomes [7,35].

A consistent picture emerged with respect to satisfaction with *Education *(opportunities to go on courses an study days/workshops). There was a sharp increase in satisfaction between 6 and 18 months across all branches. The biggest increase was for mental health nurses. Between 6 and 18 months the proportion of mental health nurses in staff nurse (E grade) positions increased from 4% to 64% compared with 0% to 11% and 0% to 32% for adult and children's nurses. There is an expectation that once someone becomes a staff nurse they should start attending post-registration courses and the fact that more mental health nurses were promoted correlates with this finding. Adjustment for moderating variables reversed the trend in satisfaction with *Education *for children's nurses so that there was now a very small increase between 6 and 18 months and a sharp fall between 18 months and 3 years (the proportion of children's nurses in staff nurse positions increased from 32% to 57%) suggesting that opportunities to attend courses had become more difficult as other responsibilities took hold and that expectations were no longer being met.

The *Development *profiles remained flat throughout the three year period although a small increase was observed between 6 and 18 months for adult branch nurses. Adjusted figures indicated a steady, but non-significant, downward trend across all three branches. Job satisfaction scores for *Development *were the second lowest (above *Pay*) for adult and child branch nurses. Nurses are therefore lacking opportunities to reflect on practice and are not receiving sufficient feedback and guidance on career development. Not having support and guidance has been identified as a reason for graduates leaving their first nursing post [1]. Higher acuity levels and inadequate nurse-to-patient ratios maybe contributing to low development scores by cutting down the time nurses have to reflect on practice and receive support.

Overall, the two branches that had the most similar findings were the adult and child branches. This perhaps was not unexpected because they have more in common with each other than they do with mental health.

The mix of censuses and samples had implications for sampling error. A census with complete information on all sampling units (nurses) will have no sampling error. Other sampling approaches may reduce sampling error by design (e.g. stratification) or increase sampling error (e.g. cluster sampling). The sampling fractions for both adult and mental branch nurses are 50% or higher and this more than compensated for any loss of precision induced by the multistage design. However we wanted to generalise findings beyond the year of survey [58] to the future and adopted a more conservative approach to sampling error by treating each population sample as a simple random sample. Additional non-sampling error may also result from non-response. This was addressed by comparing job satisfaction scores across response groups and by including variables known to predict non-response (e.g. age, gender and ethnicity) in the analysis.

Interpretation of these finding should be considered in the context of the time period in which the data were collected (1997/8 – 2000/1). Many of these findings may be as relevant as they were seven years ago although under the current climate in the NHS some of the more positive aspects that have emerged from these data may have lessened.

We end by providing some suggestions on how nurses can be supported in early career that may help improve their job satisfaction. US Research has identified a number of useful avenues that could be pursued which include providing one-year support programmes, forming new nurse support groups that meet regularly and beyond the first year and encouraging more experienced nurses to become mentors [1,9]. In the UK preceptorship is not mandatory however a formal one year preceptorship or probationary year should be considered best practice [59].

Generally supporting nurses during the transition from student to nurse will reap longer terms benefits of reduced turnover, better patient care and reduction in costs which in the UK can run as high as £10 K and result in lost productivity [53,60].

## Conclusion

We conclude that the impact of time on job satisfaction in early career is highly dependent upon specific jobs, even within the same profession. Adult, children's and mental health nurses work in different contexts and settings, often with very different organisational cultures and all of this may lead to a very different experience. Of course individuals choosing these career paths may also differ in terms of characteristics and aspirations and this also may influence the development of satisfaction. There is no single, simple answer to the trend in job satisfaction over time. Future research should focus upon understanding whether particular job characteristics could explain these findings and should not simply explore satisfaction as a unitary construct when looking at variation over time since contradictory findings emerge from different aspects of satisfaction. Further research that investigates the benefits of a formal one year preceptorship or probationary period would also come in very useful.

## Competing interests

This work was undertaken by the National Nursing Research Unit, which receives funding from the Department of Health (DH). The views expressed in this publication are those of the authors and not necessarily those of the DH.

## Authors' contributions

TM participated in study design, was involved in data processing, carried out the analysis, drafted the manuscript and the interpreted the findings. SR made a major contribution to the conception of the study, the design, data collection and interpretation. PG provided intellectual and theoretical input for the paper and interpretation of the findings. All authors were involved in revising the manuscript and have read and approved the final version.

## Pre-publication history

The pre-publication history for this paper can be accessed here:



## References

[B1] Bowles C, Candela L (2005). First job experiences of recent RN graduates. Journal of Nursing Administration.

[B2] Williams CA, Goode CJ, Krsek C (2007). Postbaccalaureate Nurse residency 1-Year Outcomes. Journal of Nursing Administration.

[B3] Robinson S, Murrells T, Clinton M (2006). Highly qualified and highly ambitious: implications for workforce retention of realising the career expectations of graduate nurses in England. Human Resource Management Journal.

[B4] Halfer D, Graf E (2006). Graduate nurse perceptions of the work experience. Nursing Economics.

[B5] Roberts BJ, Jones C, Lynn M (2004). Job satisfaction of new baccalaureate nurses. Journal of Nursing Administration.

[B6] Blegen MA (1993). Nurses job-satisfaction - A metaanalysis of related variables. Nursing Research.

[B7] Hayes LJ, O'Brien-Pallas L, Duffield C, Shamian J, Buchan J, Hughes F, Spence Laschinger HK, North N, Stone PW (2006). Nurse turnover: A literature review. International Journal of Nursing Studies.

[B8] Lu H, While AE, Barriball KL (2005). Job satisfaction among nurses: a literature review. International Journal of Nursing Studies.

[B9] Casey K, Fink R, Krugman M, Propst J (2004). The graduate nurse experience. Journal of Nursing Administration.

[B10] Fung-Kam L (1998). Job satisfaction and autonomy of Hong Kong registered nurses. Journal of Advanced Nursing.

[B11] Adams A, Bond S (2000). Hospital nurses' job satisfaction, individual and organizational characteristics. Journal of Advanced Nursing.

[B12] Porter L, Steers R (1973). Organizational, work and personal factors in employee turnover and absenteeism. Psychological Bulletin.

[B13] Morrison E, Robinson S (1997). When the employees feel betrayed: a model of how psychological contract violation develops. Academy of Management Review.

[B14] Sturges J, Guest D (2001). Don't leave me this way! A qualitative study of influences on the organisational commitment and turnover intentions of graduates in their early career. British Journal of Guidance and Counselling.

[B15] Hackman JR, Lawler EE (1971). Employee reactions to job characteristics. Journal of Applied Psychology Monograph.

[B16] Holland JL (1973). Making vocational choices: A theory of career.

[B17] Holland JL (1985). Making vocational choices: a theory of vocational personalities and work environments.

[B18] Maslow AH (1954). Motivation and Personality.

[B19] Herzberg F, Mausner B (1959). The motivation of work.

[B20] Misener TR, Haddock KS, Gleaton JU, AbuAjamieh AR (1996). Toward an international measure of job satisfaction. Nursing Research.

[B21] Kramer M (1974). Reality Shock: Why nurses Leave Nursing.

[B22] Lambert EG, Hogan NL, Barton SM (2001). The impact of job satisfaction on turnover intent: a test of a structural measurement model using a national sample of workers. The Social Science Journal.

[B23] Quinn R, Staines G (1979). The 1977 quality of employment survey: descriptive statistics with comparison data from the 1969-1970 and the 1972-73 survey.

[B24] Kovner C, Brewer C, Wu YW, Cheng Y, Suzuki M (2006). Factors associated with work satisfaction of registered nurses. Journal of Nursing Scholarship.

[B25] Loher BT, Noe RA, Moeller NL, Fitzgerald MP (1985). A meta-analysis of the relation of job characteristics to job satisfaction. Journal of Applied Psychology.

[B26] Ross CE, Reskin BF (1992). Education, control at work and job satisfaction. Social Science Research.

[B27] Spector PE (1986). Perceived control by employee: a meta-analysis of studies concerning autonomy and participation at work. Human Relations.

[B28] Roedel RR, Nystrom PC (1988). Nursing jobs and satisfaction. Nursing Management.

[B29] Acorn S, Ratner PA, Crawford M (1997). Decentralization as a determinant of autonomy, job satisfaction, and organizational commitment among nurse managers. Nursing Research.

[B30] Finn CP (2001). Autonomy: an important component for nurses' job satisfaction. International Journal of Nursing Studies.

[B31] Shoham-Yakubovich I, Carmel S, Zwanger L, Zaltcman T (1989). Autonomy, job-satisfaction and professional self-image among nurses in the context of a physicians strike. Social Science & Medicine.

[B32] Ingersoll GL, Olsan T, Drew-Cates J, DeVinney BC, Davies J (2002). Nurses' job satisfaction, organizational commitment, and career intent. Journal of Nursing Administration.

[B33] Rambur B, McIntosh B, Palumbo MV, Reinier K (2005). Education as a determinant of career retention and job satisfaction among registered nurses. Journal of Nursing Scholarship.

[B34] Blegen MA, Mueller CW (1987). Nurses job-satisfaction - A longitudinal analysis. Research In Nursing & Health.

[B35] Shields MA, Ward M (2001). Improving nurse retention in the National Health Service in England: the impact of job satisfaction on intentions to quit. Journal of Health Economics.

[B36] Clark AE, Oswald AJ (1996). Satisfaction and comparison income. Journal for Public Economics.

[B37] Department of Health (2004). Agenda for Change Final Agreement (December 2004).

[B38] Murrells T, Robinson S, Griffiths P (2008). Job satisfaction for nurses in early careers: is it the same for all branches of nursing. Journal of Nursing Management.

[B39] Marsland L, Murrells T (2000). Sampling for longitudinal study of careers of nurses qualifying from the English pre-registration Project 2000 diploma course. Journal of Advanced Nursing.

[B40] Murrells T, Clinton M, Robinson S (2005). Job satisfaction in nursing: validation of a new instrument for the UK. Journal of Nursing Management.

[B41] Spector P (1997). Job satisfaction: Application, Assessment, Cause and Consequences.

[B42] Robinson S, Marsland L, Murrells T, Hickey G, Hardyman R, Tingle A (1999). Research careers of nurse diplomates: Strategies to gain and maintain the commitment of a nationally representative cohort to a longitudinal study. Nursing Times Reseach.

[B43] Bryman A, Cramer D (1997). Quantitative Data Analysis with SPSS for Windows.

[B44] Little RJA, Rubin DB (2002). Statistical analysis of missing data.

[B45] Wolfinger R, Chang M (1995). Comparing the SAS GLM and MIXED Procedures for Repeated Measures.

[B46] Lu KY, Lin PL, Wu CM, Hsieh YL, Chang YY (2002). The relationships among turnover intentions, professional commitment, and job satisfaction of hospital nurses. Journal of Professional Nursing.

[B47] Lum L, Kervin J, Clark K, Reid F, Sirola W (1998). Explaining nursing turnover intent: job satisfaction, pay satisfaction, or organizational commitment?. Journal of Organizational Behavior.

[B48] Motowildo SJ (1983). Predicting sales turnover from pay satisfaction and expectation. Journal of Applied Psychology.

[B49] Upenieks VV (2002). Assessing differences in job satisfaction of nurses in magnet and nonmagnet hospitals. Journal of Nursing Administration.

[B50] NHS Staff Council (2005). Staff side evidence to the review body for nursing and other professional staff.

[B51] Frijters P, Shields MA, Wheatley Price S (2007). Investigating the quitting decision of nurses: panel data evidence from the British National Health Service. Health Economics.

[B52] Hutt R, Buchan J (2005). Trends in London's NHS Workforce: An updated analysis of key data.

[B53] Malhotra C (2006). Grow your own: creating the conditions for sustainable workforce development.

[B54] Holmes C (2006). The slow death of psychiatric nursing: what next?. Journal of Psychiatric and Mental Health Nursing.

[B55] Nolan P (1993). A history of mental health nursing.

[B56] Aiken L, Clarke S, Sloane D, Sochalski J, Busse R, Clarke H, Giovannetti P, Hunt J, Rafferty A, Shamian J (2001). Nurses' reports on hospital care in five countries. Health Affairs.

[B57] Aiken LH, Clarke SP, Sloane DM, Sochalski J, Silber JH (2002). Hospital nurse staffing and patient mortality, nurse burnout, and job dissatisfaction. Journal of the American Medical Association.

[B58] Kish L (1994). Multipopulation survey designs: five types with seven shared aspects. International Statistical Review.

[B59] Murrells T, Robinson S, Maben J (2008). Improving job satisfaction for newly qualified nurses. Employing Nurses and Midwives.

[B60] Select Committee on Health (2006). Evidence submitted by 5 London Strategic high-level indicators.

